# Lithocholic Acid Oleate Preparative Synthesis and Its Formulation with Lithocholic Acid as a Preventive Antiviral: In Vitro and In Vivo Assays Against HSV-1 as a Viral Infection Model

**DOI:** 10.3390/v17030416

**Published:** 2025-03-14

**Authors:** Erendira Villalobos-Sánchez, José Martín Márquez-Villa, Ana Daniela Vega-Rodríguez, David Alejandro Curiel-Pedraza, Alejandro A. Canales-Aguirre, Jorge Bravo-Madrigal, Juan Carlos Mateos-Díaz, Darwin E. Elizondo-Quiroga

**Affiliations:** 1 Medical and Pharmaceutical Biotechnology Unit, Center of Research and Assistance in Technology and Design of the State of Jalisco (CIATEJ), Guadalajara 44270, Jalisco, Mexico; endi_498@hotmail.com (E.V.-S.); dacuriel_al@ciatej.edu.mx (D.A.C.-P.); acanales@ciatej.mx (A.A.C.-A.); jbravo@ciatej.mx (J.B.-M.); 2 Industrial Biotechnology Unit, Center of Research and Assistance in Technology and Design of the State of Jalisco (CIATEJ), Zapopan 45019, Jalisco, Mexico; jmmv.ipn@gmail.com (J.M.M.-V.); advegaro@gmail.com (A.D.V.-R.)

**Keywords:** HSV-1 antivirals, preparative synthesis, lithocholic acid oleate, lithocholic acid

## Abstract

The discovery and design of antiviral agents have gained unprecedented significance due to the emergence of global health threats. The use of synthetic chemistry has enabled the modification of existing molecules and the creation of entirely novel compounds. In our laboratory, we have enzymatically synthesized a novel bioconjugate, lithocholic acid oleate (LO), derived from lithocholic acid (LCA), a bile acid that has been proven by researchers to exhibit antiviral activity in vitro. The study presented herein describes the preparative synthesis, formulation, and evaluation of LO both in vitro and in vivo for its antiviral activity against human herpes simplex virus 1 (HSV-1) as a model of viral infection. Evaluation of cytotoxicity using A549 cells indicated that a combination of LO (400 μM) and LCA (30 μM) exhibited a favorable safety profile while effectively inhibiting HSV-1 infection comparable to acyclovir treatment. Furthermore, in the in vivo assay, animals treated with an oily formulation containing 7% LO; 0.50% LCA; and 3% oleic acid (OA), 48 h prior to virus exposure, showed results even superior to a 5% acyclovir commercial formulation in terms of scar formation and wound recovery. These promising results enable the development of new preventive products against HSV-1 and probably other viruses.

## 1. Introduction

In recent years, the discovery and design of antiviral agents have gained unprecedented significance, driven by the emergence of global health threats such as novel viruses, pandemics, and the increasing prevalence of drug-resistant strains [1]. The COVID-19 pandemic, caused by the SARS-CoV-2 virus, was an evident reminder of the vulnerability of human populations to viral diseases and highlighted the urgent need for effective antiviral therapies [2]. The versatility offered by synthetic chemistry allows for the structural modification of existing molecules or the creation of entirely novel compounds [3]. One of the key advantages of synthesizing new molecules is the opportunity to discover compounds with enhanced efficacy, reduced toxicity, and improved pharmacokinetic properties [4,5].

On the other hand, bile acids have been investigated for their potential role in modulating innate immune responses, particularly in the induction of natural antimicrobial peptides (AMPs). Kong et al. demonstrated that chenodeoxycholic acid [6,7] exhibits antiviral activity against porcine delta coronavirus (PDCoV) in a porcine kidney cell line (LLC-PK1). This antiviral effect is attributed to the bile acid-induced production of interferon (IFN)-λ3 and interferon-stimulated gene 15 (ISG15), key components of the cellular antiviral defense mechanism [8]. Also, Liang et al. demonstrated that bile acids, such as chenodeoxycholic acid (CDCA), stimulate antiviral response in hepatic cells, against different RNA viruses including vesicular stomatitis virus (VSV), human influenza virus A/H1N1 (IAV), and encephalomyocarditis virus (EMCV) [9].

Bile acids have been employed in synthetic chemistry to create conjugates with diverse bioactive compounds, enhancing properties such as bioavailability, reducing cytotoxicity, and improving drug delivery efficiency [10]. In our laboratory, we have synthesized various molecules derived from LCA; one of them is the lithocholic acid oleate (LO); this molecule increases the expression of human beta-defensin 1 (HBD1) and Cathelicidin LL37 in human keratinocytes [11]; both AMPs have recognized antimicrobial capacity [12] and, in some studies, they also show antiviral activity [9,13].

One of the most infectious viruses is the human herpes simplex virus type 1 (HSV-1), which is an enveloped, double-stranded DNA virus, belonging to the Herpesviridae family and the simplex virus genus [14]. Globally, 3.8 billion people under the age of 50 (64.2%) are infected with the virus. Once infected, individuals may experience lifelong outbreaks triggered by various factors such as emotional stress, sun exposure, and other stimuli [15]. Although three classes of drugs are currently approved for the treatment of HSV targeting viral DNA replication (acyclic guanosine analogs, acyclic nucleotide analogs, and pyrophosphate analogs), drug resistance to these antivirals is well documented [16,17]. Therefore, the search for new treatments, especially those that could stimulate a response generated by the host, is of great importance.

In the study, presented herein, a novel bioconjugate, LO, was enzymatically synthesized from LCA and oleic acid (OA), then conveniently formulated and evaluated in vitro and in vivo for its antiviral activity against human herpes simplex virus 1 (HSV-1) as a model of viral infection.

## 2. Materials and Methods

### 2.1. Cells and Virus

Human Alveolar Basal Epithelial (A549) cells (CRM-CCL-185 ™), and African green monkey kidney epithelial (Vero) cells (ATCC^®^ CCL-81 ™), were purchased from the American Type Culture Collection (ATCC, Manassas, VA, USA) and grown with Dulbecco’s Modified Eagle Medium (DMEM) supplemented with 2 mM L-glutamine and 10% (*v*/*v*) fetal bovine serum (FBS, Gibco-Life Technologies, Carls-band, CA, USA). For stimulation assays, the medium was switched to DMEM supplemented with 1% FBS. Cells were maintained at 37 °C in a humidified atmosphere containing 5% CO_2_.

The HSV-1 (VR-1789 ™) also was purchased from the ATCC; viral stocks were prepared by infecting Vero cell monolayers with HSV-1 in DMEM. After a 72 h incubation period, the cells were lysed through a freeze–thaw cycle and centrifuged at 1500× *g* for 20 min at 4 °C. The resulting supernatant was collected, filtered using a 0.20 µm membrane, and stored at −80 °C for subsequent analysis.

### 2.2. Compounds

LCA (CAS 434-13-9), OA (CAS 112-80-1), 2-methyl-2-butanol (CAS 75-95-85), 2-methyl-2-propanol (CAS 78-83-1), tetrahydrofuran (CAS V000241), dimethyl sulfoxide (DMSO) (CAS 67-68-5), hexane (CAS 110-54-3), ethyl acetate (CAS 141-78-6), 2,2,4-trimethylpentane (CAS 540-84-1), petroleum ether (CAS 64742-49-0, Merck KGaA, Darmstadt, Germany), ethanol (CAS 64-17-5), acetic acid (CAS 64-19-7), phosphomolybdic acid (CAS 51429-74-4), KMnO_4_ (CAS 7722-64-7), NaOH (CAS 1310-73-2), K_2_CO_3_ (CAS 584-08-7), phosphomolybdic acid (CAS 51429-74-4), molecular sieves 3 Å (CAS 308080-99-1), recombinant Lipase B from *Candida antarctica* immobilized on Immobead 150 (CAS 52583), and TLC aluminum plates with fluorescence silica gel (CAS Z193291-1PAK) were purchased from Sigma-Aldrich (St. Louis, MO, USA). For the preparative synthesis, industrial-grade LCA (CAS-434-13-9, PI Chemicals Ltd., Shanghai, China) and OA (CAS-112-80-, Napamex, Zapopan, Mexico) were also employed. For the formulation for in vivo assays, cosmetic grade coco caprylate (CAS 61788-47-4), castor oil (CAS 8001-79-4), benzyl alcohol (CAS 100-51-6), and tocopheryl acetate (CAS 7695-91-2) were purchased from Gardenia Naturals (Guadalajara, Mexico).

Acyclovir for the antiviral in vitro experiments was purchased from Sigma Aldrich (CAS 59227-89-3), and for the in vivo experiments, acyclovir cream was purchased at the pharmacy (Cicloferon^®^ crema para fuegos, Liomont, Ciudad de Mexico, Mexico; batch number: J02062).

### 2.3. Lithocholic Acid Oleate Reaction Solvent Screening

LO reaction solvent screening was performed in 40 mL amber vials (IKA, Staufen, Germany) containing 20 mL of 100 mM LCA and 150 mM of OA, using different polar solvents (i.e., 2-methyl-2-propanol, tetrahydrofuran, dimethyl sulfoxide, and 2-methyl-2-butanol). Then, 50 mg/mL of immobilized *Candida antarctica* B lipase as biocatalyst and 50 mg/mL of 3 Å molecular sieves were added to each vial. The reaction mixture was maintained at 50 °C with magnetic stirring (500 RPM) for 120 h. After incubation, the product formation was qualitatively monitored using thin-layer chromatography (TLC).

### 2.4. Lithocholic Acid Oleate Preparative Synthesis and Purification

LO preparative synthesis was performed in a 200 mL V2 vessel SpinChem Rotating Bed Reactor (RBR) (SpinChem, Umeå, Sweden) containing 150 mM of LCA and 100 mM of OA, in 150 mL of 2-methyl-2-butanol. Then, 25 mg/mL of immobilized *Candida antarctica* B lipase as biocatalyst and 25 mg/mL of 3 Å molecular sieves were loaded into the rotating bed. The reaction mixture was maintained at 50 °C with stirring (500 RPM) for 48 h. After incubation, the reaction mixture was removed from the rotating bed reactor and the catalyst recovered. 2-methyl-2-butanol was removed from the mixture using a rotary evaporator (Heidolph, Schwabach, Germany). The LO and the remaining substrates were fully concentrated for further purification. The concentrate was redissolved in petroleum ether, cooled overnight at −20 °C, and then filtered to remove the remaining LCA. Product formation over time and the purified LO were qualitatively monitored/analyzed using thin-layer chromatography (TLC). The purified LO was stored at −20 °C for further assays.

### 2.5. Lithocholic Acid Oleate Formulation for In Vivo Assays

For in vivo assays, a mixture of coco caprylate/castor oil//benzyl alcohol/tocopheryl acetate (1/1/0.125/0.125) was employed as a diluent of the antiviral molecules. This diluent without antiviral molecules was employed as a control. Three formulations of different LO concentrations mixed with OA and LCA in the diluent were prepared and called T0 = diluent; T = 1 (1.75% LO; 0.75% OA and 0.125% LCA); T = 2 (3.5% LO; 1.5% OA and 0.250% LCA); T = 3 (7% LO; 3% OA and 0.50% LCA).

### 2.6. Thin-Layer Chromatography and High-Performance Thin-Layer Chromatography

The migration of the samples was performed in a climatic test chamber (CAMAG, Muttenz, Switzerland) using hexane/ethyl acetate/acetic acid (20/10/0.3) as the mobile phase. TLC plates were revealed using an oxidizing solution composed of 0.75% (*w*/*v*) KMnO_4_, 0.056% (*w*/*v*) NaOH, and 10% (*w*/*v*) K_2_CO_3_. Alternatively, a solution of 10% (*w*/*v*) phosphomolybdic acid in absolute ethanol was employed. TLC plates were sprayed using a glass laboratory sprayer with a rubber bulb (Macherey-Nagel, Allentown, PA, USA).

LO final conversión of the preparative synthesis and purity were quantified/analyzed by High-Performance Thin Layer Chromatography (HPTLC). All samples were conveniently diluted in isooctane/2M2B (9/1) and loaded using a Linomat 5 (CAMAG, Muttenz, Switzerland) for automatic and accurate sample dosing and analyzed using the winCATS software. LCA/OA standard curves and samples were migrated and revealed as previously described and spots were scanned at 500 nm with a Scanner 3 (CAMAG, Muttenz, Switzerland).

### 2.7. Virus Titration

Virus titration was conducted using the TCID_50_ assay. Ten-fold serial dilutions of the virus were prepared and used to infect Vero cell monolayers pre-cultured in a 96-well plate. The medium was removed from the plate, and 100 µL of each virus dilution was added to the cells. The plate was incubated for 72 h at 37 °C with 5% CO_2_. Wells showing cytopathic effects (CPE) were counted for each dilution. The 50% infectious dose TCID_50_/mL was calculated using the Reed and Muench method [18].

### 2.8. Maximal Non-Cytotoxic Dose (MNCD)

To find the maximal non-cytotoxic dose (MNCD) of the different compounds that were used in the antiviral assays as treatments, the cytotoxicity was assessed by examining their effects on cell morphology and cell viability, using A549 cells. A known antiviral drug, acyclovir (Sigma-Aldrich, St. Louis, MO, USA) was included in the assay. Stock solutions at 0.2 M were prepared in dimethyl sulfoxide and stored at −20 °C. Each compound was diluted in DMEM immediately before use.

Cell monolayers were cultured in 96-well plates and exposed to varying concentrations of the compounds, ranging from 20 to 500 µM, for 72 h. Cytotoxicity was estimated based on morphological alterations, such as cell rounding, shrinkage, and detachment. The cell viability was determined using a tetrazolium-based colorimetric assay with 3-(4,5-dimethylthiazol-2-yl)-2,5-diphenyltetrazolium bromide (MTT, Sigma). 100 µL for a well of MTT was added to the cells at a concentration of 5 mg/mL, prepared in phosphate-buffered saline (PBS). After a 2 h incubation period, the resulting formazan crystals were dissolved in 100 µL of isopropanol. The optical density (OD) was then measured at 570 nm using a microplate reader. The maximal non-cytotoxic dose was selected at which cell viability remained ≥80% with no observable morphological changes detected by microscopic monitoring. The 50% cytotoxic concentration (CC50) was determined as the concentration that reduced absorbance by 50% compared to the negative control (cells without treatment). These values were estimated using probit analysis of the dose–response generated from the experimental data.

### 2.9. Cytopathic Effect Inhibition Assays

To evaluate the potential antiviral capacity of the compounds, A549 cells were seeded at a density of 1 × 10^5^ cells per well, in 24-well plates using two different approaches: a simultaneous treatment assay and a pre-treatment assay. In both approaches, treatments included the MNCD of OA, LO, LCA, or a combination of LO and LCA. Acyclovir (20 µM) was used as a positive drug control, while untreated cells served as the infection control for HSV-1.

In the simultaneous assay, the MNCD of each treatment was mixed with 50 TCID_50_/mL of HSV-1 and immediately added to the A549 cell monolayers. The plates were then incubated for 72 h until CPE observations. In the pre-treatment assay, the MNCD of each treatment was applied to the cells for 24 h before viral infection. After this incubation, the treatment supernatants were removed, and 50 TCID_50_/mL of HSV-1 was added to the monolayer for viral adsorption. Following a 2 h incubation, the treatment supernatants were reintroduced. In a separate set of experiments, a fresh medium containing the treatments were added instead. In both cases, the plates were incubated for 72 h until CPE observations.

For both approaches, CPE progression was monitored using light microscopy. The antiviral effects of the tested compounds were evaluated based on the presence or absence of CPE. Supernatants were collected, pooled, and stored at −80 °C until further use.

### 2.10. Antiviral Plaque Reduction Assay

We conducted a viral plaque reduction assay to assess the inhibitory effect of the compounds on HSV-1 infection in A549 cells. The 24-well plates were seeded to achieve the monolayer formation of A549 cells. The plates were then emptied and washed twice with 1X PBS to remove any residual media or non-adherent cells. Next, the MNCD of each compound was added to the cells, and the plates were incubated for 2 h at 37 °C with 5% CO_2_. Then, media supernatants were removed and 50 TCID_50_/mL of HSV-1 was added to the monolayer for viral adsorption. Following a 2 h incubation, the inoculum was removed and replaced with 1 mL of overlay medium (a 1:1 dilution of 3% carboxymethyl cellulose in 2X DMEM with 2% FBS, along with previously recovered supernatants). The plates were incubated for 72 h in a 5% CO_2_ atmosphere at 37 °C. After incubation, the overlay was decanted, and the wells were washed three times with 1X PBS to eliminate any residual semi-solid medium. The plaques were then fixed and stained with 1% crystal violet in methanol for 15 min at room temperature. After staining, the solution was removed, and the monolayers were rinsed with water until the plaques became visible.

### 2.11. Viral Yield-Reduction Assay

The supernatants recovered from the pre-treatment antiviral assay were titrated using the TCID_50_ assay to assess the residual infectivity. For each compound, 100 µL of supernatant was used to prepare 10-fold serial dilutions in DMEM. Using a 96-well plate pre-seeded with A549 cells, the medium was discarded, and serial dilutions of the supernatants were added to the wells in eight replicates. The plates were incubated at 37 °C for 48 h until CPE became apparent. Subsequently, the wells showing positive CPE were counted, and the viral titer was calculated using the Reed and Muench method [18].

### 2.12. Quantitative qPCR

To quantify the viral genomic copies of HSV-1 after the treatment, total viral DNA was isolated from the culture cell supernatants in triplicates using the KIT Biospin Virus DNA/RNA extraction kit (BioFlux, Hangzhou, China) according to the manufacturer’s instructions. Quantitative real-time PCR was performed using QuantiFast Sybr Green qPCR KIT (Qiagen). The primer sequences used to detect HSV-1 were as follows: HSV-1 forward primer 5′-GACTCTCCCACCGCCATCAG-3′ and HSV-1 reverse primer 5′-TGTCTTCGGGCGACTGGT-3′ [19]. The amplification was carried out in a LightCycler 480 II PCR instrument (Roche Diagnostics, Penzberg, Germany) for 40 cycles at 95 °C for 15 s and 60 °C for 1 min. For absolute quantitation of the viral DNA, a standard curve was established with a serially diluted in vitro transcribed DNA of HSV-1 with a known copy number.

### 2.13. In Vivo Study

For the animal model, Nu/Nu male mice (4–5 weeks old) were purchased from Bioterios de la Facultad de Ciencias, Universidad Nacional Autonoma de Mexico (Ciudad de Mexico, Mexico), and used for the experiments. Animals were quarantined for 10 days before the start of the experiments. All the animals were kept at 26 °C, with a 12 h night/day cycle, and supplied with a laboratory rodent diet (Safe U8027G10R) and water ad libitum. Because HSV-1 is unable to penetrate intact skin, the infection model requires compromising skin integrity through scarification [20]. To prevent struggling or pain, the animals were anesthetized intraperitoneally with ketamine (5.0–7.5 mg/kg). Scarification was performed using a technique adapted from Goel et al., 2002, in which the skin was damaged with a single, small, and shallow scratch using a 30 G needle [21]. After scarification, animals were inoculated with HSV-1 cultured in Vero cells, by rubbing the area for 10 s with a cotton-tipped applicator [22] soaked in a medium containing 1 × 10^6^ TCID_50_/mouse of HSV-1 (in a 50 µL volume).

Adapted from Duan et al., 1998, all topical treatments were applied 3 h after inoculation and continued daily for 20 days (four times a day between 8:30 a.m. and 5:30 p.m.) [23]. However, in the T4 group, treatment began 48 h before virus inoculation and was subsequently administered following the same schedule. Animals were photographed daily throughout the study to monitor lesion progression caused by viral replication. The following criteria were applied to score topical lesions: 0, no lesion; 1, discrete vesicles; 2, two or more open lesions; 3, separate ulcerations; and 4, Zoster band formations. Animals were housed in standard polycarbonate mice cages with filtered air units adapted to the ventilated racks (RAIG model 17093) with an *n* = 5 per group. All groups except for the control group were infected with 1 × 10^6^ virus per mouse (Table 1). As a drug control, a separate group was treated with commercial acyclovir cream.

The study was approved by the Center for Research and Assistance in Technology and Design of the State of Jalisco (CIATEJ) Biosafety Committee (Third Ordinary session, August 2023, number: CBS-2023-3-1). Animal procedures were approved by the CIATEJ CICUAL Code 2023-021A.

### 2.14. Statistics

The 50% cytotoxic concentrations (CC50) were determined using GraphPad PRISM 8.0.2 software (Graph-Pad Software, San Diego, CA, USA). Values of *p* < 0.05 were considered indicative of statistical differences. * *p* < 0.05, ** *p* < 0.01, *** *p* < 0.005, and **** *p* < 0.001. Results represent the mean ± SEM of two independent experiments (*n* = 3).

## 3. Results

### 3.1. Lithocholic Acid Oleate Reaction Solvent Screening

**Figure 1:**
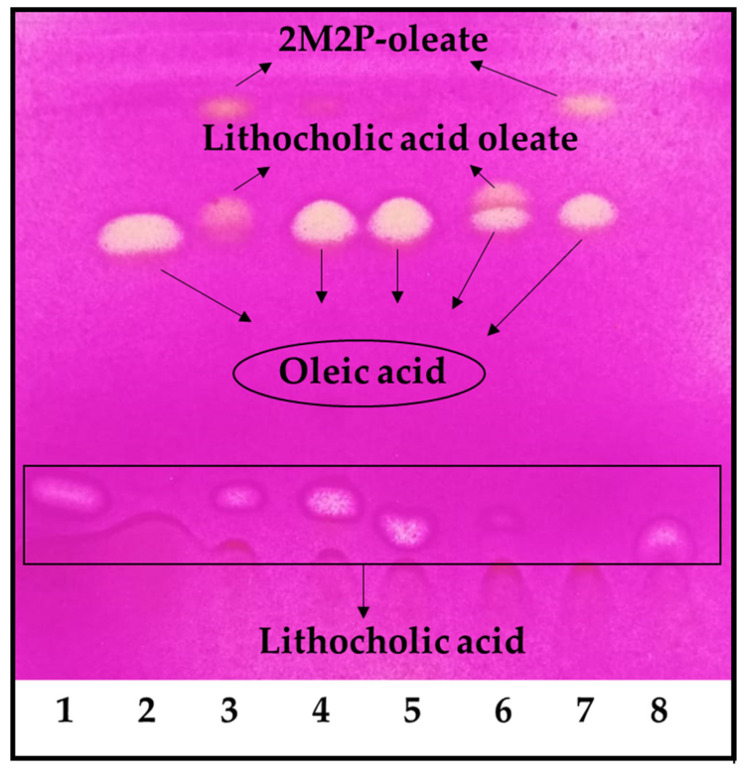
LO reaction solvent screening. 1. LCA, 2. OA, 3. Reaction in 2-methyl-2-propanol, 4. Reaction in tetrahydrofuran, 5. Reaction in dimethyl sulfoxide, 6. Reaction in 2-methyl-2-butanol, 7. Control reaction of OA in 2-methyl-2-propanol, 8. Control reaction of LCA in 2-methyl-2-propanol. The reaction was performed using an OA molar excess: 100 mM of LCA and 150 mM of OA.

Under the reaction conditions employed, the highest conversion was achieved with 2-methyl-2-butanol and 2-methyl-2-propanol, but the reaction did not occur in tetrahydrofuran and dimethyl sulfoxide (Figure 1). Using 2-methyl-2-propanol, the appearance of an undesirable by-product decreased the final conversion. It was demonstrated that the by-product was 2-methyl-2-propanol oleate (Figure 1, lines 3 and 7). Thus, 2-methyl-2-butanol was selected as the reaction solvent for the LO preparative synthesis. It is worth noting that these reactions were carried out using an OA molar excess; thus, the remaining OA should be removed during purification. The first purification attempt was carried out under these conditions; however, purification yields were low (<10%). Thus, we decided to use LCA in molar excess, to facilitate the LO purification in the preparative synthesis.

### 3.2. Lithocholic Acid Oleate Preparative Synthesis and Purification

**Figure 2:**
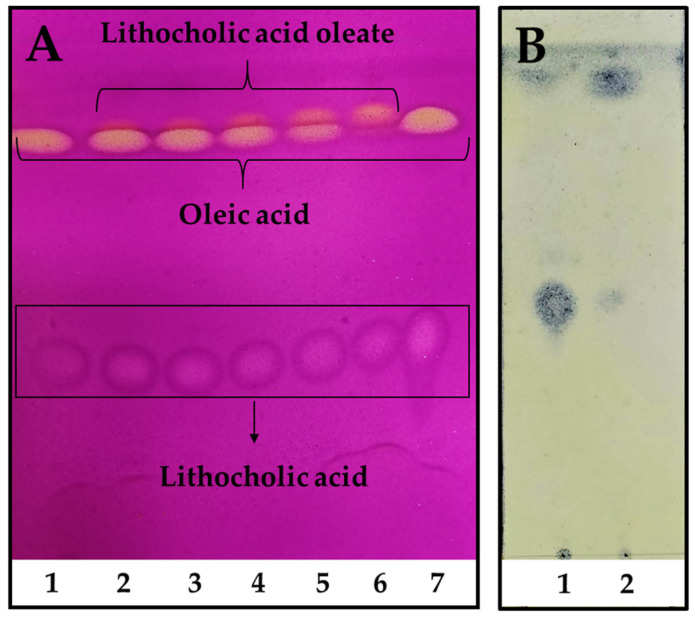
Reaction kinetics of LO preparative synthesis and purification. (**A**). Reaction kinetics time 1. 0 h, 2. 6 h, 3. 8 h, 4. 12 h, 5. 24 h, and 6. 48 h, 7. LCA and OA standards. The reaction was performed using a molar excess of LCA: 150 mM of LCA and 100 mM of OA. TLC was revealed using 0.75% (*w*/*v*) KMnO_4_, 0.056% (*w*/*v*) NaOH, and 10% (*w*/*v*) K_2_CO_3_. (**B**). LO purification. 1. Remaining LCA after petroleum ether extraction, 2. Purified LO extracted with petroleum ether. TLC was revealed using 10% (*w*/*v*) phosphomolybdic acid in absolute ethanol.

In order to scale up the reaction, industrial pharmaceutical-grade reactants were employed for the preparative enzymatic synthesis and product purification. Moreover, a fully scalable rotatory bed reactor was employed, which enables a seamless transition from laboratory to full-scale industrial processes. The reaction reached its highest conversion after only 48 h, with almost a total consumption of OA used as a limiting reagent (Figure 2A, line 6). As seen in Figure 2B, LCA was easily and almost totally removed from the reaction mixture after petroleum ether extraction, leaving the LO only with OA traces. The final LO yield after purification was ~66%, allowing to obtain around 40 g of product/L of reaction.

**Figure 3:**
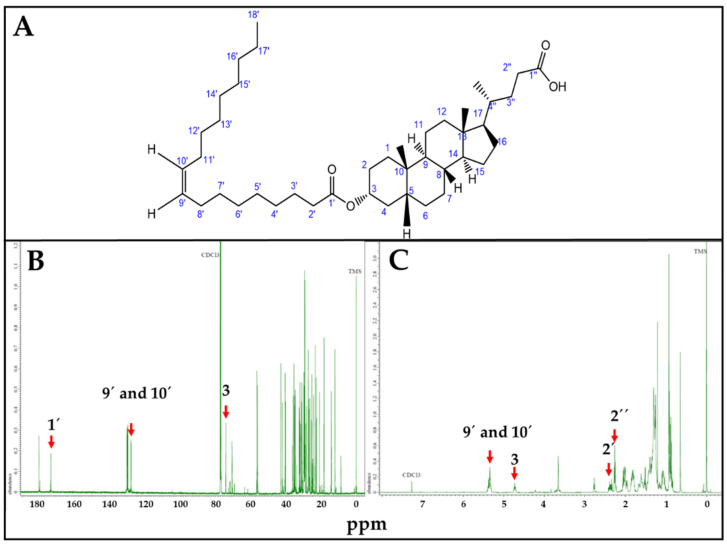
LO, NMR spectra. (**A**). LO structure. (**B**). Carbon 13 (^13^C) spectrum. 1′ = 173.1 ppm; 9′ and 10′ = 130.6 ppm; 3 = 73.9 ppm. (**C**). Proton (^1^H) NMR spectrum. 9′ and 10′ = 5.34 ppm; 3 = 4.61 ppm; 2′ = 2.35, 2″ = 2.33 ppm. ppm values corresponding to characteristic ^13^C and ^1^H NMR peaks were estimated using Chemdraw.

LO synthesis was confirmed by NMR (Figure 3). Characteristic LO Carbon 13 (Figure 3B) and proton (Figure 3C) NMR peaks were correlated with estimated ppm values, using Chemdraw. In the Carbon 13 (^13^C) NMR spectrum, 1′ = 173.1 ppm corresponds to the carboxylic carbon of OA; 9′ and 10′ = 130.6 ppm to the sp2 carbons of the OA double bond, and 3 = 73.9 ppm to the carbon linked to LCA-OH group. Similarly, for Proton (^1^H) NMR spectrum, 9′ and 10′ = 5.34 ppm correspond to the hydrogens linked to the sp2 carbons of the OA double bond; 3 = 4.61 ppm to the hydrogen linked to the carbon linked to LCA-OH group; 2′ = 2.35 ppm and 2″ = 2.33 ppm to the hydrogen linked to the alpha carbon of OA and LCA carboxylic functional groups, respectively.

### 3.3. Maximum Non-Cytotoxic Dose

**Figure 4:**
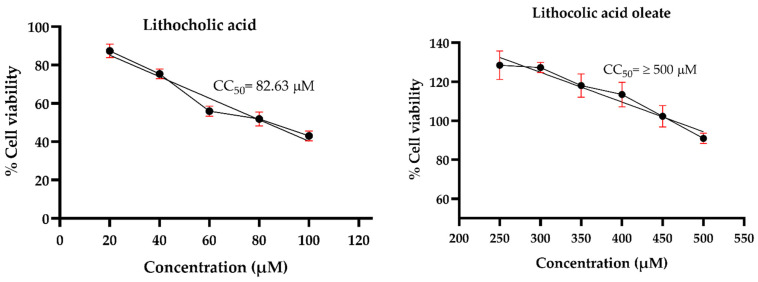
Cytotoxicity of LO and LCA in A549 cells. The cytotoxicity (CC50) was expressed as the concentration at which 50% of the cells died in DMEM containing varying concentrations of the tested molecules. Graphs show the mean values of two experiments (*n* = 4). The error bars represent the standard deviation.

Prior to evaluating the antiviral effects against HSV-1, an MNCD assay was conducted to determine the appropriate concentrations of the synthetic conjugates and their precursors in A549 cells. Morphological changes were examined through microscopic observations, while cell viability was assessed using the MTT assay. The results revealed that LO exhibited lower cytotoxicity compared to its precursor, LCA. The precursor LCA and the conjugated LO induced significant morphological changes at concentrations of 40 µM and 450 µM, respectively. Although LO 450 µM maintained cell viability above 80%, noticeable morphological alterations were observed. Therefore, concentrations of 30 µM for LCA and 400 µM for LO were selected for subsequent antiviral activity assays against HSV-1. Acyclovir (Sigma^®^) was tested at concentrations of 10, 20, 30, 40, 50, and 60 µM to determine the maximum non-cytotoxic concentration (MNCD) and the 50% cytotoxic concentration (CC_50_). The CC_50_ was calculated to be 59.54 µM, while 20 µM was identified as the MNCD and selected for further experiments (Figure 4). Additionally, OA did not induce morphological changes at concentrations above 250 µM, and non-cytotoxic effects were observed in cells treated with 0.1% DMSO.

### 3.4. Assessment of Anti-HSV-1 Activity in A549 Cells

**Figure 5:**
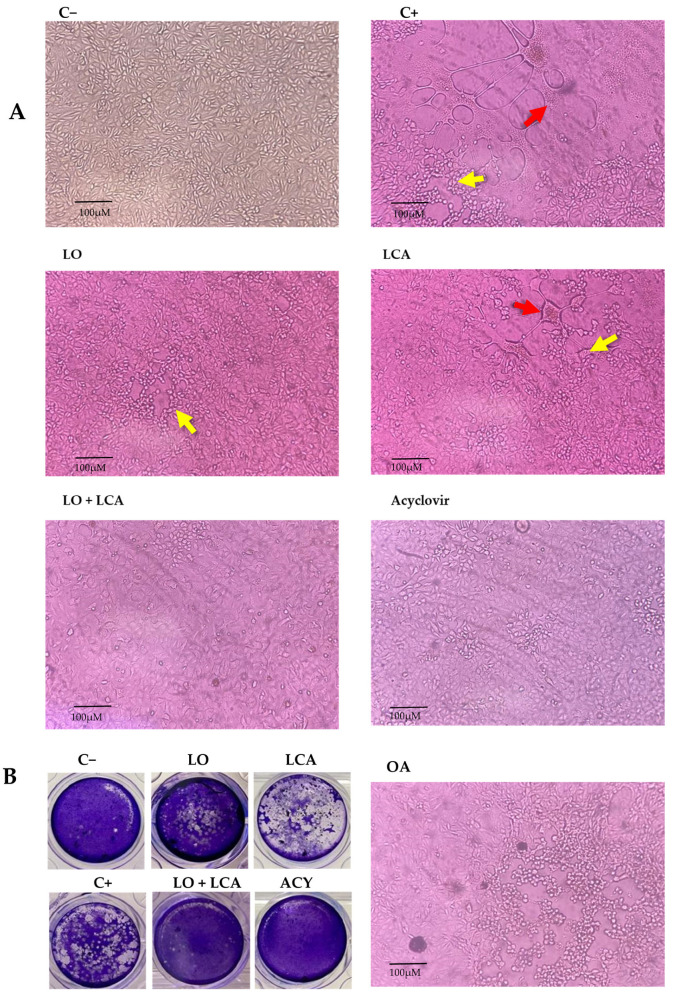
Inhibition of HSV-1 in A549 cells. (**A**): Representative photographs from the cytopathic effect (CPE) inhibition assay at 72 h post-infection. A549 cells were pretreated for 24 h with different treatments and then infected with HSV-1. C−: Uninfected A549 cells; C+: A549 cells infected with HSV-1, showing CPE characterized by cell detachment (yellow arrow) and syncytia formation (red arrow). LO: A549 cells treated with 400 µM LO; LCA: A549 cells treated with 30 µM LCA; LO + LCA: A549 cells treated with 400 µM LO in combination with 30 µM LCA; acyclovir: A549 cells treated with 20 µM acyclovir; OA: A549 cells treated with 250 µM oleic acid (original magnification 10×). (**B**): Inhibition of HSV1 by viral plaque reduction assay. The images show plaque formation due to HSV-1 infection (C+) and the absence or reduction in plaques following treatment. A significant reduction in plaque formation was observed with LO or LCA treatment, while complete inhibition was achieved with the LO + LCA combination, as well as with acyclovir treatment.

Using the MNCD of the compounds, experiments were conducted to evaluate their antiviral activity against HSV-1 in vitro using A549 cells. To assess this, a CPE inhibition assay was performed at two-time points: simultaneous and pre-treatment. Treatments included LO, LCA, OA, and the LO + LCA mix, with acyclovir (20 µM) as a positive drug control, while untreated cells served as the infection control for HSV-1. Each treatment was tested in four replicates. In the simultaneous assay, none of the treatments provided protection against the virus, as CPE appeared in all replicates when the compounds and HSV-1 were introduced simultaneously, except for the acyclovir control, where no CPE was observed. The same results as in the simultaneous assay were observed in the case where A549 cells were pretreated for 24 h with the different treatments, and a fresh medium was added. However, when A549 cells were pretreated for 24 h with the different treatments and treated supernatants were reintroduced after the virus absorption period, all the treatments significantly reduced CPE. The most pronounced effect was observed with the LO + LCA combination, where no signs of CPE were detected in any replicate. Similarly, no CPE was observed in the acyclovir control. LO treatment resulted in minimal CPE characterized by small areas of cell rounding and detachment without syncytia formation. LCA treatment reduced the overall CPE areas in the cultures; however, syncytia formation was still observed. In contrast, the HSV-1 positive control exhibited extensive CPE (infected cells displaying detachment, multinucleated giant cells, and syncytia). OA did not protect cell cultures from HSV-1 infection (Figure 5A).

To evaluate the inhibitory effect on viral replication, a plaque reduction assay was performed for LO, LCA, and LO + LCA and acyclovir in the pre-treatment condition, as this was the only assay that demonstrated CPE inhibition. The LO + LCA treatment exhibited a potent inhibitory effect against HSV-1, with no viral plaques detected, indicating complete suppression of CPE. Similarly, no viral plaques were detected in the acyclovir treatment. In contrast, LO and LCA as separate treatments resulted in an uncountable number of plaques. Untreated cells showed extensive monolayer destruction due to viral replication (Figure 5B).

To assess the differences in viral load among treatments, supernatants were collected to determine the residual viral titer using the TCID_50_ assay. As shown in Figure 5A, no significant differences were observed between the LCA treatment and the positive control. LO treatment reduced the viral load by 1.24 Log_10_ in comparison to the positive control. Additionally, the combination of LCA and LO resulted in a more significant reduction in viral load, comparable to acyclovir treatment, when compared to the positive control of HSV-1 infection. The differences were statistically significant, with a *p*-value < 0.001.

**Figure 6:**
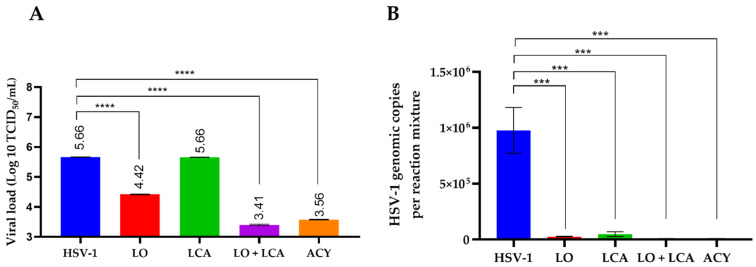
Infectious viral yield reduction assay (**A**) and genomic copies (**B**) obtained from qPCR detection. **A**: Results are plotted as the log₁₀ of the viral yield, calculated using the TCID_50_ method. HSV-1 represents the untreated, HSV-1-infected cultures. **** *p* < 0.0001, *** *p* < 0.0005 one-way analysis of variance (ANOVA) with Tukey’s multiple comparisons test when compared with the untreated control. **B**: Data are presented as the mean of genomic copies quantified using q-PCR from three replicates ± SD.

Virus titration revealed that the highest inhibitory effect on viral growth was observed with the combination of LO + LCA, as well as with acyclovir treatment. To correlate these results, residual viral DNA was detected using q-PCR. Real-time PCR demonstrated the presence of viral DNA after 72 h post-infection in both treated and untreated cells. However, the results showed that the LO + LCA combination significantly reduced the levels of viral DNA (Figure 6B). Overall, these results exhibited a remarkable inhibitory effect on viral replication.

### 3.5. In Vivo Model

Since antiviral activity against HSV-1 was observed during the in vitro assays, with a more significant reduction in viral load in the LO + LCA combination, an in vivo experimental model was designed to test this mixture. Seven experimental groups of Nu/Nu mice (*n* = 5 per group) were used, assessing three different concentrations of LO + LCA (T1, T2, and T3) for topical application. A fourth group (T4) received the highest concentration of LO + LCA, applied 48 h prior to virus inoculation. Topical acyclovir was used as a drug control (T5), while an HSV-1-infected group treated only with diluent was included as the positive control (T0). The seventh group was the negative control group (mice without treatment).

**Figure 7:**
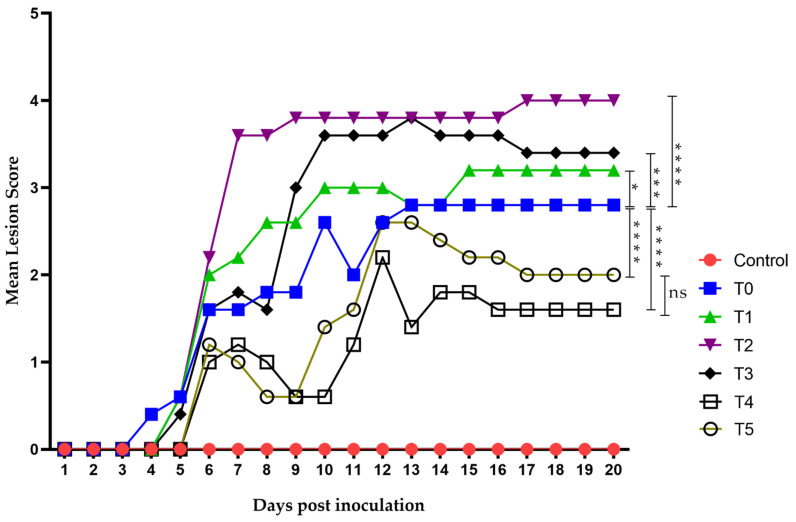
Time evolution of the mean lesion score. Lesion scoring was performed using a nominal scale based on clinical presentation as follows: 0, no lesion; 1, discrete vesicles; 2, two or more open lesions; 3, separate ulcerations; and 4, Zoster band formations. The first lesions appeared on day 4 post-inoculation, with all groups exhibiting some form of lesion by day 6. Group T2 showed the worst clinical outcome, whereas groups T4 and T5 had the least severe clinical presentations. Zoster band formation was observed on day 7 post-inoculation in group T2 and on day 12 in group T5. Overall, groups T4 and T5 demonstrated the best clinical recovery, characterized by improved outcomes and faster healing. The area under the curve (AUC) was analyzed using Graphad Prism 8. Groups were compared using ANOVA analysis with a post hoc test, Sydak’s multiple comparisons test. **** *p* < 0.0001, *** *p* < 0.0002, * *p*< 0.01 and ns: no statistical differences. There was no statistically significant difference in AUC values between the T5 group (Acyclovir) (25.40 ± 4.21) and T4 group (group pre-treated with LO + LCA 48 h before virus inoculation) (20.00 ± 3.44).

Virus inoculation in mice was performed using 1 × 10^6^ TCID_50_/mouse of HSV-1 (in a 50 µL volume), and the animals developed topical lesions as expected. The first lesions appeared 96 h post-inoculation as discrete vesicles, becoming more evident at 120 h in all groups except the negative control. The first group to develop lesions was T0 (the group receiving only the diluent without treatment), followed by T1 to T3 between days 4 and 5. Groups T4 (treated 48 h before virus inoculation) and T5 (acyclovir-treated) showed lesions by day 6 post-inoculation. Most groups began developing herpes zoster-like lesions seven days after exposure, and all groups eventually exhibited such lesions. The final lesions appeared in groups T4 and T5 on days 12 and 13, respectively, coinciding with their peak lesion scores (Figure 7).

Groups T1 to T3 did not show a strong response against the virus, with their maximum mean lesion scores ranging between 3 and 4 throughout the experiment. In contrast, group T4 showed results even superior to acyclovir, with a maximum mean lesion score of 2 on day 12, which decreased to 1 from day 16 onwards. Group T5 (acyclovir-treated) showed a maximum mean lesion score of 2.5 on days 12 and 13, maintaining a score of 2 until day 19, and decreasing to 1.5 on day 20.

**Figure 8:**
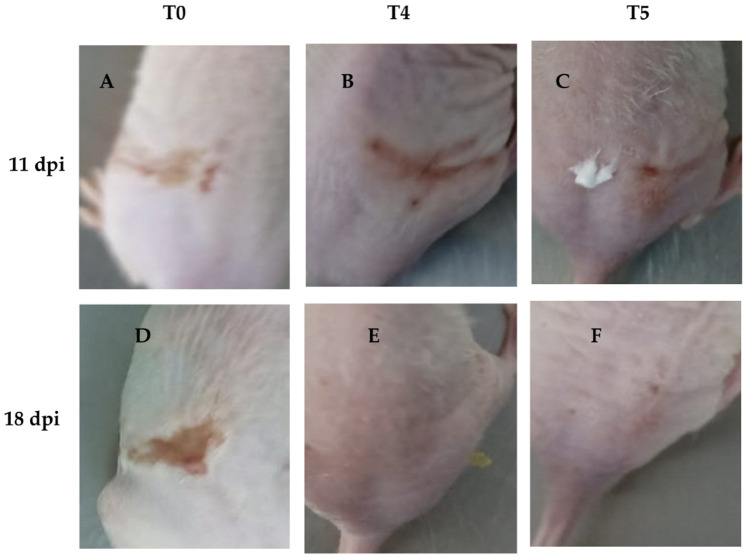
Representative images of groups T4 and T5 showing infection recovery during the experiment. We found that wound healing was more effective in group T4 compared to T5, with a noticeable reduction in scar formation and improved skin repair. (**A**): HSV-1 infected mice without treatment at 11 dpi; (**B**): HSV-1 infected mice and treated with LO + LCA at 11 dpi; (**C**): HSV-1 infected mice treated with acyclovir at 11 dpi; (**D**): HSV-1 infected mice without treatment at 18 dpi; (**E**): HSV-1 infected mice and treated with LO + LCA at 18 dpi; (**F**): HSV-1 infected mice treated with acyclovir at 18 dpi.

On the other hand, wound recovery and re-epithelialization in the T4 group were superior compared to the other groups (Figure 8B,E). In contrast, the T1 and T2 groups showed no significant effect on wound recovery, exhibiting poor healing characterized by fibrosis and skin folding, pretty similar to the control group (T0) (Figure 8A,D). Although the T3 group did not demonstrate better clinical outcomes compared to the other concentrations, it showed slightly improved performance. In contrast, acyclovir (T5) exhibited better clinical outcomes in terms of HSV skin pathology (Figure 8C,F), but did not result in superior wound healing compared to T4, nor did it show as good re-epithelialization as T4.

## 4. Discussion

To our knowledge, this is the first enzymatic synthesis and formulation report of LO as an antiviral. LO (Figure 3) is an ester bioconjugate of LCA and OA with a molecular weight of 640 g/mol. Its chemical synthesis under general esterification conditions (i.e., H_2_SO_4_; HCl; DCC or EDC/DMAP) needs LCA carboxylic moiety protection/deprotection [24,25,26], since it may react with its -OH group leading to polymerization. Moreover, alcohols can not be employed as reaction solvents under chemically catalyzed conditions, because they may react with OA. Thus, chemoselective enzymatic synthesis using immobilized *Candida antarctica* lipase was chosen for LO synthesis. As shown in Figure 1, different solvents, including ternary alcohols, were tested. Interestingly, the reaction only occurred using 2-methyl-2-propanol (2M2P) and 2-methyl-2-butanol (2M2B) as reaction solvents, albeit with different results. Despite their similar structure and physicochemical properties, 2M2P leads to the formation of the undesirable 2M2P-oleate by-product, whereas 2M2B does not.

On the other hand, initial purification yields using an OA molar excess during the reaction were low because column chromatography was needed to separate LO from the remaining OA. However, when using a LCA molar excess, its removal from petroleum ether by simple filtration was possible, and LO was easily recovered after solvent vacuum evaporation (Figure 2B), allowing the preparative downstream processing and obtaining enough product for in vitro and in vivo assays.

To determine the antiviral effect against HSV-1, cells and the virus were treated with the MNCD of the compounds during infection. The findings of this study indicate that the bioconjugate LO exhibits lower cytotoxicity compared to its precursor, LCA, in A549 cells in our in vitro assays. When testing antiviral activity, we first demonstrated that LO, when used as a pretreatment, exhibits antiviral activity against HSV-1. Therefore, considering its low cytotoxicity and antiviral activity, LO could be a promising candidate for further antiviral development. The lower cytotoxicity and increased antiviral activity observed with LO are consistent with previous research in which the conjugation of 3-oxo-lithocholic acid with N-methylpiperazine was synthesized. This modification led to a dramatic increase in antiviral activity against the AH1N1 virus, along with a two-fold reduction in toxicity in MDCK cells [27].

Interestingly, the combination of LO + LCA in a pre-treatment assay, when treated supernatants were reintroduced after the virus absorption period, significantly reduced the CPE of the viral infection. Moreover, in the plaque reduction assay, the LO + LCA combination provided complete protection against the virus, as no viral plaques were detected. In contrast, LO or LCA alone resulted in uncountable plaque formation, indicating incomplete viral inhibition. Other studies have found that unconjugated bile acids, CDCA and LCA, inhibit delta coronavirus replication in vitro [8]. Although in our study LCA did not exhibit the strongest antiviral activity on its own, its activity could be enhanced when combined with other molecules or chemically conjugated, as shown in the study by Petrova et al., against influenza A/H1N1 virus [27].

Additionally, our results from the residual viral load measurement by TCID50 revealed a substantial reduction in viral titers, with the LO + LCA combination lowering the titer to levels comparable to acyclovir treatment. Also, q-PCR data from our study supported these results by demonstrating a low viral load in cells treated with LO + LCA, indicating a delay in viral replication.

These in vitro results suggest three important points: (1) The inhibition of HSV-1 appears to be mediated by the stimulation of antiviral peptides in the cells, as CPE inhibition was observed only in the pre-treatment assay where the stimulated supernatants were reintroduced to the cell cultures. This is particularly important since bile acids have been shown to increase the expression of AMPs [12,28]; it remains to be determined which molecules are secreted in the presence of LO + LCA and how they might act against other viruses. (2) LO, when used as a pretreatment, exhibits antiviral activity against HSV-1. Nevertheless, the LO + LCA combination appears to be superior to either compound alone, as it significantly reduced viral infection according to results obtained from CPE inhibition, the plaque reduction assay, and qPCR data. (3) A high concentration of LCA is required to achieve the antiviral effect, which could be facilitated through bioconjugation with oleic acid. Future studies will be needed to isolate, detect, and characterize the active molecules present in the stimulated medium. Importantly, our results were comparable to acyclovir treatment in all assays. No CPE was detected, and the residual viral yield was similar, with no statistically significant differences.

In vitro assays demonstrated a synergy between LO and LCA, thus an adequate antiviral topical formulation containing LO/LCA at a 14/1 (*w*/*w*) proportion, was necessary to incorporate both actives in a monophasic oily base. LCA must be first solubilized in benzyl alcohol and the mixture then incorporated into the LO with the other ingredients (i.e., coco caprylate, castor oil, and tocopheryl acetate) to obtain a stable monophasic formulation for in vivo assays.

The T4 group (pre-treated with LO + LCA) exhibited delayed lesion onset (similar to acyclovir) and lower maximum lesion scores, suggesting a potential prophylactic effect of the combination. In contrast, the T3 group (same concentration as T4 but applied 3 h after inoculation) did not show strong protection. This suggests that if treatment is applied appropriately (Pretreatment), it may yield enhanced antiviral effects.

The effect observed in T4 on wound healing could be explained by two components: the first could be due to a decrease in the HSV-1 viral load from the treatment, and the second could be related to components of the formulation that modulate the pro-inflammatory response. A common phenomenon in wound healing is the acute phase response, characterized by elevated levels of inflammatory markers. It has been reported that the regulation of these elements contributes to improved wound repair. Notably, LCA has been shown to inhibit IL-17A production [29]. IL-17A is known to activate key transcription factors such as NF-κB, which regulates the expression of various inflammatory cytokines and chemokines [20,30]. Additionally, IL-17A influences the MAPK pathways, which are critically involved in wound-healing processes [31]. Another component in our formulation that may contribute to wound healing is α-tocopherol (α-TA), due to its antioxidant capacity and its role in the reprogramming of gene expression related to inflammatory processes in the skin [32]. Additionally, benzyl alcohol in our formulation has been associated with the TGF-β/Smad signaling pathway, which has been implicated in the inhibition of fibroblast proliferation, promotion of fibroblast apoptosis, blockage of fibroblast differentiation into myofibroblasts, and suppression of collagen synthesis [33,34,35]. The treatment significantly enhanced the wound healing process during the recovery phase of infection in the T4 group compared to T5, leading to a noticeable reduction in scar formation and improved skin repair. This effect may be attributed to both the reduction in viral load and the specific components of the formulation. Interestingly, the T0 group, which received the diluent containing all formulation components except for the active molecules (LO + LCA), did not exhibit wound healing. This outcome suggests that ongoing viral replication likely hindered the diluent’s ability to facilitate skin recovery.

In contrast, while the T5 group (Acyclovir group) displayed antiviral activity, it did not achieve the same level of wound resolution as the T4 group, particularly in terms of scar reduction and skin repair. This observation suggests that post-viral skin recovery following antiviral treatment may primarily depend on the host’s intrinsic healing capacity rather than the antiviral itself.

## 5. Conclusions

LO was successfully synthesized via enzymatic catalysis on a multigram preparative scale using a fully scalable rotary bed reactor, facilitating the transition from laboratory to full industrial-scale production. Our study supports the preventive antiviral efficacy of both LO and LCA, particularly when used in combination against HSV-1. In vitro assays demonstrated a synergistic antiviral effect of the LO + LCA combination against HSV-1, with results comparable to acyclovir treatment across all pre-treatment assays. Consequently, an effective oily formulation incorporating both active compounds was developed for in vivo testing. Moreover, when applied prior to viral infection in mice, this formulation outperformed the commercial acyclovir formulation under recommended usage conditions, potentially enabling the development of new commercial preventive products against HSV-1. Ongoing studies are also being conducted with other viruses, yielding promising results.

## Figures and Tables

**Table 1:** Experimental mice groups and different treatments.

Group Designation	Treatment	Concentrations
Control group	Mice without treatment	N/A
T0	Group of HSV-1 (positive control) mice with diluent	Diluent: coco caprylate/castor oil/benzyl alcohol/tocopheryl acetate (1/1/0.125/0.125) (*w*/*w*)
T1	Mice treated with LO + LCA dose 1 (T 1.75%)	Diluent + 1.75% lithocholic acid oleate, 0.75% oleic acid, and 0.125% lithocholic acid
T2	Mice treated with LO + LCA dose 2 (T 3.5%)	Diluent + 3.5% lithocholic acid oleate, 1.5% oleic acid, and 0.250% lithocholic acid
T3	Mice treated with LO + LCA dose 3 (T 7%)	Diluent + 7% lithocholic acid oleate; 3% oleic acid and 0.50% lithocholic acid
T4	Mice treated with LO + LCA dose 3, 48 h before virus inoculation	Diluent + 7% lithocholic acid oleate; 3% oleic acid and 0.50% lithocholic acid
T5	Mice treated with acyclovir cream	N/A

## Data Availability

Data are contained within the article.
